# Structural model of health-promoting behavior in middle-aged Korean women with hypertension based on the Information–Motivation–Behavioral Skills model: a cross-sectional study

**DOI:** 10.4069/whn.2026.05.27

**Published:** 2026-06-30

**Authors:** Eunju Mun, Jinhwa Park

**Affiliations:** 1 Department of Nursing, Suseong University, Daegu, Korea; 2 College of Nursing, Daegu Catholic University, Daegu, Korea

**Keywords:** Health promotion, Hypertension, Information motivation behavioral skills model, Middle aged, Women

## Abstract

**Purpose:**

This study aimed to develop and evaluate a hypothetical model of health-promoting behaviors among middle-aged women with hypertension, guided by the Information–Motivation–Behavioral Skills (IMB) framework. The endogenous variables were self-efficacy in chronic disease management and health-promoting behaviors, and the exogenous variables were hypertension-related knowledge, eHealth literacy, health attitudes, and social support.

**Methods:**

This cross-sectional study used an online structured questionnaire. A total of 331 middle-aged women diagnosed with hypertension participated. Data were analyzed using IBM SPSS ver. 25.0 and IBM SPSS AMOS ver. 25.0 to test the hypothesized model and evaluate model fit.

**Results:**

The proposed model showed satisfactory fit to the data. Of the nine hypothesized pathways, six were statistically significant. Hypertension-related knowledge, social support, and self-efficacy in chronic disease management had significant direct effects on health-promoting behaviors. In contrast, eHealth literacy and health attitudes did not have statistically significant direct effects on health-promoting behaviors. Health attitudes and social support had significant indirect and total effects on health-promoting behaviors. The model explained 60.4% of the variance in health-promoting behaviors, indicating the importance of hypertension-related knowledge, health attitudes, social support, and self-efficacy in chronic disease management.

**Conclusion:**

The findings suggest that the proposed IMB-based model may help explain health-promoting behaviors among middle-aged women with hypertension. The results indicate that self-efficacy in chronic disease management may link motivational factors to behavioral outcomes and may inform future intervention research aimed at promoting health behavior change in this population.

## Introduction

Hypertension is a major global public health concern, and its prevalence has increased alongside population aging [1]. When coronavirus disease 2019-related expenditures are excluded, hypertension accounts for the largest single-disease expenditure in the Korean national health insurance system [2]. Among Korean adults in their 40s and 50s, more than one-third have been diagnosed with hypertension. Although the overall prevalence of hypertension is higher in men than in women across the life course, prevalence increases more rapidly in women than in men [3].

For women, midlife typically begins in the 40s; by the 50s, menopause-related hormonal changes increase the risk of hypertension [4] and its complications [5]. Among middle-aged women, hypertension is the leading risk factor for both cardiovascular disease incidence and cardiovascular mortality [4], and hypertension-related mortality is approximately twice that of middle-aged men [6]. Early diagnosis and timely treatment are essential for preventing hypertension-related cardiovascular damage [1]. Among middle-aged women with hypertension, greater engagement in health-promoting behaviors contributes to more stable blood pressure management [6] and reduces the likelihood of hypertension-related health problems [5]. Supporting these behaviors is therefore important for healthy aging and for maintaining quality of life in later years [7].

In South Korea, middle-aged women have been described as prioritizing the health of spouses, children, and other family members over their own. This family-centered orientation has been associated with relatively lower engagement in health-promoting behaviors [8]. Consistent with this sociocultural context, middle-aged women with hypertension have also been reported to have insufficient engagement in health-promoting behaviors [9]. Supporting health-promoting behaviors among middle-aged women with hypertension therefore requires identification of multidimensional factors that reflect the characteristics of this population.

The Information–Motivation–Behavioral Skills (IMB) model provides a useful framework for examining these factors. The IMB model is an established theoretical framework for understanding and promoting health behavior [10]. It posits that people are most likely to initiate and sustain beneficial health behaviors and achieve positive health outcomes when they have accurate information, are motivated to act, and possess the skills needed to perform the behaviors [11].

Within the IMB framework, the information component includes disease knowledge [12,13] as well as understanding of one’s health status and the consequences of health behaviors [14]. Disease-related knowledge can help individuals adopt healthier lifestyles [15], yet many people with hypertension still lack adequate or accurate information about their condition [16]. eHealth literacy, defined as the capacity to locate, interpret, and assess health information available on the internet [17], has been identified as a predictor of health-promoting behaviors [18]. In the IMB model, eHealth literacy functions as an informational determinant [19,20]. Individuals with higher eHealth literacy may be better able to use online resources appropriately [21] and may be more likely to engage in health-promoting activities [22]. Access to and use of online health information among middle-aged women have increased; however, much of the information available on websites is unreliable, incomplete, or difficult to understand [21]. Further research is therefore needed to examine how different aspects of information, including eHealth literacy, are associated with health-promoting behaviors in this group.

The motivation component includes health attitudes [15] and social support [13]. Positive health attitudes in patients with hypertension are associated with stable blood pressure control [16] and greater readiness to adopt health-promoting behaviors [15]. Previous studies have reported that individuals with chronic diseases tend to have more negative health attitudes than healthy adults [9,15]. Social support from family members, friends, neighbors, and healthcare providers also promotes health-promoting behaviors, with higher perceived support associated with greater behavioral engagement [23].

Within the behavioral skills component, self-efficacy [12,15] is a key determinant of adopting and maintaining healthy lifestyle practices among individuals with hypertension [24]. Among middle-aged women with hypertension, greater self-efficacy is associated with fewer perceived barriers and a higher likelihood of behavioral change [25], resulting in more consistent engagement in health-promoting behaviors [23].

Previous IMB-based studies have used structural equation modeling (SEM) to identify factors related to health-promoting behaviors in populations such as patients with schizophrenia [26] and patients with heart failure [27]. However, research focusing specifically on middle-aged women with hypertension remains limited. Although numerous studies have examined factors associated with health-promoting behaviors among middle-aged women [7,28], limitations remain in translating these findings into effective intervention strategies for middle-aged women with hypertension. In particular, previous findings regarding psychosocial factors [15,26] and eHealth literacy [29] have been inconsistent, and the interrelationships among informational, motivational, and behavioral skills components have not been sufficiently examined within an integrated theoretical framework.

Therefore, this study examined the pathways among information, motivation, behavioral skills, and health-promoting behaviors among middle-aged women with hypertension, based on the IMB model and prior findings. The study also sought to provide foundational evidence for developing nursing interventions to support health-promoting behaviors in this population.

**Figure 1.**
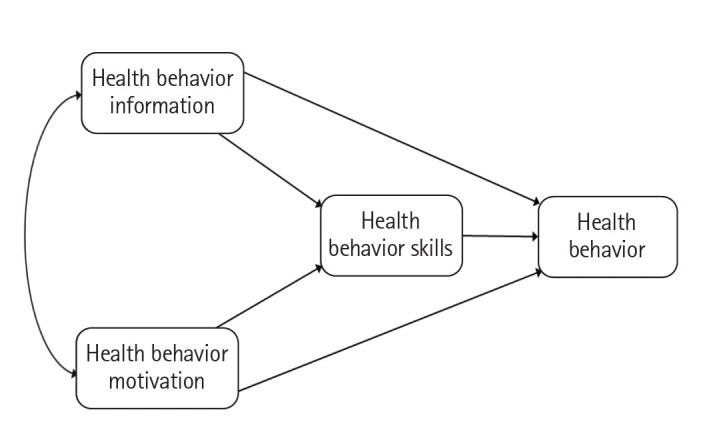
Theoretical model within the Information–Motivation–Behavioral Skills model proposed by Fisher and Fisher [11].

The conceptual framework was developed from the IMB model proposed by Fisher and Fisher [11] and prior empirical research to examine the structural relationships among variables associated with health-promoting behaviors in middle-aged women with hypertension. The rationale for each proposed pathway is described below (Figure 1).

**Figure 2.**
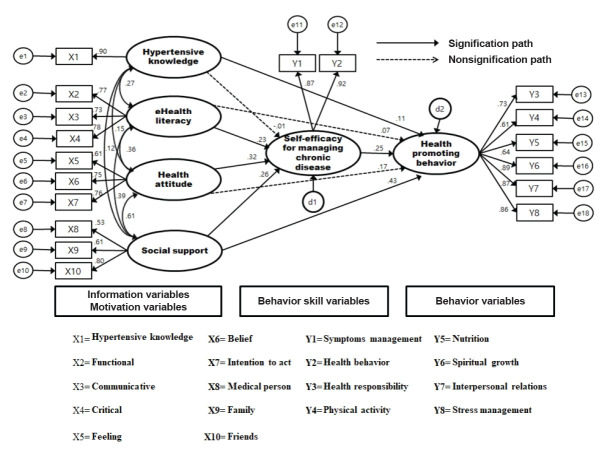
Path diagram of the hypothetical model.

First, disease-related knowledge was hypothesized to be associated with self-efficacy because greater understanding of a disease may increase confidence in managing one’s condition [12,30]. Accordingly, hypertension-related knowledge was specified as having a direct pathway to self-efficacy in chronic disease management in the proposed model. In addition, hypertension-related knowledge has been reported to be associated with health-promoting behaviors [23]. Therefore, a direct pathway from hypertension-related knowledge to health-promoting behaviors was specified on theoretical and empirical grounds. Second, although eHealth literacy has been conceptualized as a multidimensional construct that includes behavioral skills in previous studies [31], it was positioned within the information component of the IMB model in this study. Within the IMB framework, the information component encompasses accurate knowledge about specific health behaviors, awareness of one’s health status, and understanding of the consequences of engaging or not engaging in those behaviors [32,33]. Accordingly, eHealth literacy was conceptualized as an informational capacity, based on prior empirical findings [19,20] and the theoretical definition of information in the IMB model. eHealth literacy may be associated with self-efficacy by supporting individuals’ ability to access, evaluate, and apply credible health information [30], and it may also be indirectly associated with health-promoting behaviors through self-efficacy [18]. Some studies have also reported a direct association between eHealth literacy and health-promoting behaviors [22,34]. Third, health attitudes were conceptualized as a motivational factor that may be associated with self-efficacy [15] and indirectly associated with health-promoting behaviors through self-efficacy [27,28]. Health attitudes have also been reported to have direct associations with health-promoting behaviors [35]. Fourth, social support was also regarded as an important motivational resource. Perceived support from family members, peers, and healthcare providers has been associated with engagement in health-promoting behaviors [23] and with self-efficacy [26]. It has also been associated with health-promoting behaviors indirectly through self-efficacy [13]. Finally, self-efficacy and related behavioral skills have been consistently associated with engagement in health-promoting behaviors [28]. Figure 2 presents the research model derived from the literature review and study hypotheses. Both direct and mediated pathways were theoretically specified in the proposed model rather than explored in an ad hoc manner.

## Methods

**Ethics statement:** This study was approved by the Institutional Review Board of Daegu Catholic University (No. 2024-0015). Informed consent was obtained from the participants.

### Study design

This cross-sectional online survey was conducted to develop and test a hypothetical model of health-promoting behaviors among middle-aged women diagnosed with hypertension. The study followed the STROBE (Strengthening the Reporting of Observational Studies in Epidemiology) reporting guidelines (http://www.strobe-statement.org) in describing the methods and findings.

### Participants

Participants were middle-aged women with hypertension in South Korea. The inclusion criteria were as follows: (1) age 40–64 years [6,7], (2) diagnosis of hypertension by a physician, and (3) full awareness of the study purpose and procedures. The exclusion criterion was hospitalization or residence in a long-term care facility at the time of data collection.

According to established guidelines, an appropriate sample size for SEM using maximum likelihood estimation ranges from 200 to 400 participants, or approximately 10 to 20 times the number of observed variables [36]. Because this study included 18 observed variables—one factor for hypertension-related knowledge, three for eHealth literacy, three for health attitudes, three for social support, two for self-efficacy in chronic disease management, and six for health-promoting behaviors—a sample size of 200 to 360 was considered appropriate. To account for potential dropout or data exclusion, data collection continued until 347 responses were obtained through the online survey. After 16 responses with insincere or invalid entries were excluded, the final sample comprised 331 participants (95.4%).

### Measurements

#### Hypertension-related knowledge

Hypertension-related knowledge was assessed using the Hypertension Knowledge Scale developed by Min and Hur [37]. The scale consists of 20 items, with 1 point assigned for each correct response and 0 points assigned for incorrect and “I don’t know” responses. Total scores range from 0 to 20, with higher scores indicating greater hypertension-related knowledge. Reliability was not reported in the original study [37]. In the present study, the Kuder–Richardson 20 (KR-20) coefficient was .51.

#### eHealth literacy

eHealth literacy was measured using the eHealth Literacy Scale developed by Lee [38]. The instrument includes 31 items across three subdomains: functional eHealth literacy (eight items), communicative eHealth literacy (11 items), and critical eHealth literacy (12 items). Items are rated on a 5-point Likert scale. Total scores range from 31 to 155, with higher scores indicating greater eHealth literacy. In this study, scores were converted to mean scores for analysis. In Lee’s original study [38], Cronbach’s α values were .90, .92, and .93 for the three subdomains, respectively. In this study, the overall Cronbach’s α was .96, with subdomain reliabilities of .93, .94, and .91, respectively.

#### Health attitudes

Health attitudes were measured using the Health Attitude Scale, originally developed by Torabi and Jeng [39] and later translated and adapted by Kim [40]. The scale comprises 15 items across three subdomains: emotional responses to health (five items), beliefs about disease prevention and healthy lifestyles (five items), and behavioral intentions toward health practices (five items). Items are rated on a 5-point Likert scale. Five items (items 1, 4, 5, 10, and 13) were reverse-coded. Total scores range from 15 to 75, with higher scores indicating more positive health attitudes. In this study, scores were converted to mean scores for analysis. In the original study [39], the overall Cronbach’s α was .88, with subdomain reliabilities of .85, .74, and .85, respectively. In Kim’s adaptation [40], the overall Cronbach’s α was .84, with subdomain reliabilities of .66, .68, and .61, respectively. In the present study, the overall Cronbach’s α was .83, with subdomain reliabilities of .64, .64, and .86, respectively.

#### Social support

Social support was measured using the Multidimensional Scale of Perceived Social Support (MSPSS), developed by Zimet et al. [41] and translated into Korean by Shin and Lee [42]. The MSPSS comprises 12 items across three subdomains: family support (four items), friend support (four items), and significant other support (four items). Items are rated on a 5-point Likert scale. Total scores range from 12 to 60, with higher scores indicating greater perceived social support. In this study, scores were converted to mean scores for analysis, and “significant other” specifically referred to support from healthcare providers. In the study by Zimet et al. [41], Cronbach’s α ranged from .84 to .92 across populations, and in the study by Shin and Lee [42], it was .89. In the present study, the overall Cronbach’s α was .90, with subdomain reliabilities of .94, .90, and .88, respectively.

#### Self-efficacy in chronic disease management

Self-efficacy in chronic disease management was assessed using the Korean version of the Shortened Chronic Disease Management Self-Efficacy Scale (SECD-6-K), translated and validated by Kim et al. [43]. The SECD-6-K comprises six items in two subdomains: symptom management (two items) and health behavior (four items). Items are rated on a 10-point scale. Total scores range from 6 to 60, with higher scores indicating greater self-efficacy in chronic disease management. In this study, scores were converted to mean scores for analysis. In the study by Kim et al. [43], the overall Cronbach’s α was .96, with subdomain reliabilities of .96 and .92, respectively. In the present study, the overall Cronbach’s α was .96, with subdomain reliabilities of .91 and .97, respectively.

#### Health-promoting behavior

Health-promoting behavior was measured using the Health-Promoting Lifestyle Profile II (HPLP-II), developed by Walker et al. [44] and translated into Korean by Seo and Hah [45]. The HPLP-II consists of 50 items across six subdomains: health responsibility (eight items), physical activity (eight items), nutrition (nine items), spiritual growth (nine items), interpersonal relations (eight items), and stress management (eight items). Items are rated on a 4-point Likert scale. Total scores range from 50 to 200, with higher scores indicating greater engagement in health-promoting behaviors. In this study, scores were converted to mean scores for analysis. In the study by Seo and Hah [45], the overall Cronbach’s α was .92. In the present study, the overall Cronbach’s α was .96, with subdomain reliabilities of .85, .91, .77, .89, .87, and .79, respectively.

### Data collection

Institutional review board approval was obtained on July 5, 2024, and data collection was conducted thereafter. The survey was administered online between July 5 and August 5, 2024, using a structured questionnaire. Approval was obtained from workplace and residential administrators before recruitment announcements were distributed. Data were also collected simultaneously through an online survey company using the same questionnaire. Individuals who reviewed the notice and voluntarily expressed interest received a unique link to the questionnaire. Only participants who selected “I consent” could proceed to the survey. To prevent duplicate participation, the survey platform was configured to allow only one entry per user account, and no duplicate IP addresses were detected during data review. After completing the survey, participants received a mobile gift certificate worth 5,000 Korean won as a token of appreciation.

### Data analysis

All analyses were conducted using IBM SPSS Statistics ver. 25.0 and ver. AMOS 25.0 (IBM Corp., Armonk, NY, USA). Descriptive statistics, including frequencies, percentages, means, standard deviations (SDs), skewness, and kurtosis, were used to summarize participant characteristics and assess distributional assumptions. Internal consistency of the measurement tools was evaluated using Cronbach’s α coefficients.

Confirmatory factor analysis was performed to evaluate the construct validity of the latent variables. SEM with maximum likelihood estimation was then conducted in AMOS to assess the hypothesized model. Path significance was determined using regression weights, standardized estimates, standard errors, critical ratios, and *p*-values, and explanatory power was examined using squared multiple correlations.

Model fit was evaluated using the following fit indices and criteria: *χ*², *χ*²/df, goodness-of-fit index (GFI), adjusted goodness-of-fit index (AGFI), root mean square residual (RMR), standardized root mean square residual (SRMR), root mean square error of approximation (RMSEA), incremental fit index (IFI), Tucker-Lewis index (TLI), and comparative fit index (CFI). The significance of direct, indirect, and total effects in the modified model was tested using bootstrapping.

## Results

### General characteristics of participants

The general characteristics of the participants are presented in Table 1. The mean age was 53.34 years (SD=6.29 years), and nearly half of the participants were in their 50s. Most participants lived with family members (n=305, 92.1%) and were employed (n=242, 73.1%). The mean duration of hypertension was 7.70 years (SD=6.63 years), and most participants were taking antihypertensive medication (n=304, 91.8%). A total of 91 participants (27.5%) had hypertension only. The most common comorbidities were dyslipidemia (n=158, 47.7%), obesity (n=136, 41.1%), and diabetes mellitus (n=47, 14.2%).

Subjective health status was reported as healthy by 63 participants (19.1%), moderate by 145 participants (43.8%), and unhealthy by 123 participants (37.1%). A total of 207 participants (62.5%) had experienced menopause, including 33 (10.0%) with surgical menopause and 174 (52.6%) with natural menopause. The mean age at menopause was 51.42 years (SD=4.38 years). In addition, 71 participants (21.5%) reported regular menstruation and 53 (16.0%) reported irregular menstruation. Most participants drank alcohol no more than once per month (n=242, 73.1%), followed by 3 to 4 times per month (n=53, 16.0%), 1 to 2 times per week (n=24, 7.3%), and at least three times per week (n=12, 3.6%). There were 19 current smokers (5.7%) and 312 non-smokers (94.3%).

### Descriptive statistics of measured variables and correlations

Descriptive statistics for the study variables are presented in Table 2. Except for the Hypertension Knowledge Scale, all instruments demonstrated satisfactory reliability. The internal consistency of the Hypertension Knowledge Scale was evaluated using the KR-20 coefficient because of its dichotomous response format, yielding a coefficient of .51, which is below the generally accepted threshold of .60. A low KR-20 coefficient suggests that an instrument may not adequately capture a unidimensional construct [46]. The Hypertension Knowledge Scale assesses five domains: general knowledge of blood pressure, influencing factors, treatment, medication, dietary control, and exercise and stress management. This domain structure indicates that the scale is multidimensional [37]. Despite its low reliability, the scale was retained in the hypothetical model based on previous studies demonstrating its usefulness in populations similar to that of the present study and within the IMB framework [11,37].

Normality was examined using means, SDs, skewness, and kurtosis. All absolute skewness and kurtosis values were within ±3, supporting the assumption of univariate normality. Therefore, maximum likelihood estimation was used for parameter estimation [36]. Multicollinearity was assessed using correlation coefficients, tolerance values, and variance inflation factor (VIF) values. All variables were significantly and positively correlated, with coefficients ranging from r=.11 to r=.79 (*p*<.05). Tolerance values ranged from .70 to .95, exceeding the minimum criterion of .10, and VIF values ranged from 1.05 to 1.43, well below the cutoff of 10. These findings indicate that multicollinearity was not a concern [47].

### Validity of the measurement model

Convergent validity indicates the extent to which indicators intended to measure the same construct are consistent with one another [36]. In this study, all measurement paths were significant, with critical ratios ranging from 8.31 to 19.90, exceeding the criterion of 1.96. Standardized factor loadings ranged from .53 to .92, which was within the acceptable range of .50 to .95. Construct reliability values ranged from .94 to 1.00, exceeding the recommended minimum of .70, and average variance extracted (AVE) values ranged from .89 to .97, exceeding the criterion of .50. Collectively, these findings support the convergent validity of the constructs [36]. Hypertension-related knowledge was not included in the construct reliability or factor analyses because it reflected knowledge across specific domains rather than a latent construct.

Discriminant validity concerns whether latent constructs are empirically distinct [36]. In this study, squared correlation coefficients ranged from .01 to .53, and all AVE values exceeded the squared correlations, supporting discriminant validity. Because the Hypertension Knowledge Scale was scored as a single total value, construct reliability and AVE values could not be calculated for this variable. Instead, discriminant validity was examined using correlation coefficients and standard errors [27], which indicated adequate discriminant validity among the constructs.

Nomological validity evaluates whether observed associations among constructs are consistent with the hypothesized theoretical framework [36]. In this study, nomological validity was supported because the observed correlations aligned with the predicted directions.

### Validity of the path model

The hypothesized path model was specified based on the IMB model [11] and previous empirical findings. Model fit was evaluated using multiple goodness-of-fit indices. Because the hypothesized model demonstrated acceptable fit and was consistent with the theoretical framework, it was retained as the final model.

The final goodness-of-fit indices for the hypothesized path model were as follows: *χ*²=400.42 (*p*<.001), *χ*²/df=3.31, AGFI=.82, CFI=.90, IFI=.91, RMR=.03, SRMR=.05, and RMSEA=.08 (Table 3). Because the *χ*² statistic is highly sensitive to sample size and tends to reach significance in larger samples, it should not be interpreted in isolation [46]. Instead, several fit indices should be considered, including RMSEA, for which values of .05 to .08 indicate acceptable fit; IFI and CFI, for which values ≥.90 indicate good fit; and RMR and SRMR, for which values <.08 indicate adequate fit [36]. Because GFI may be inflated in models with more variables and greater complexity, AGFI was also reported. An AGFI value ≥.80 is generally considered acceptable [47]. Although the overall fit indices met commonly accepted criteria, the RMSEA (.08) and AGFI (.82) values indicated only marginal adequacy. Thus, the model should be considered acceptable but not optimal, and the structural relationships should be interpreted with caution.

### Effect analysis of the path model

The direct, indirect, and total effects of the exogenous variables on the endogenous variables are presented in Table 3 and Figure 2. Hypertension-related knowledge was not associated with self-efficacy in chronic disease management (β=–.01, *p*=.806), but it was associated with health-promoting behavior (β=.11, *p*=.024). eHealth literacy was associated with self-efficacy in chronic disease management (β=.23, *p*=.012), but it was not associated with health-promoting behavior (β=.07, *p*=.418). Health attitudes were associated with self-efficacy in chronic disease management (β=.32, *p*=.017), but they were not associated with health-promoting behavior (β=.17, *p*=.125). However, health attitudes were indirectly associated with health-promoting behavior through self-efficacy in chronic disease management (β=.08, *p*=.023), and their total effect was statistically significant (β=.24, *p*=.048). Social support was associated with self-efficacy in chronic disease management (β=.26, *p*=.006) and health-promoting behavior (β=.43, *p*=.001). Social support was also indirectly associated with health-promoting behavior through self-efficacy in chronic disease management (β=.07, *p*=.012), and its total effect was statistically significant (β=.49, *p*=.001). Finally, self-efficacy in chronic disease management was associated with health-promoting behavior (β=.25, *p*=.007). These variables explained 60.4% of the variance in health-promoting behavior, and six of the nine hypothesized paths were statistically significant.

## Discussion

This study developed and tested a structural model to explain health-promoting behaviors among middle-aged women with hypertension, drawing on the IMB model [11] and previous empirical findings. First, hypertension-related knowledge was significantly associated with health-promoting behaviors among middle-aged women with hypertension. This finding is consistent with previous research showing that greater hypertension-related knowledge is associated with higher levels of health-promoting behaviors [1]. However, previous studies have also indicated that knowledge may vary according to educational attainment [10] and disease duration [1], suggesting that information alone may have limited association with behavioral engagement [23]. Accordingly, educational approaches that incorporate practical, disease-specific management strategies may be more relevant than approaches focused solely on basic knowledge, although this requires further investigation.

Hypertension-related knowledge was not significantly associated with self-efficacy in chronic disease management. This finding differs from previous studies showing that exercise knowledge among patients with heart failure [27] and osteoporosis knowledge among patients with osteoporosis [12] were associated with self-efficacy. According to the IMB model, when a target behavior requires complex skills, information may influence behavior indirectly through behavioral skills rather than being directly associated with self-efficacy [10]. Previous research has suggested that once individuals’ knowledge reaches a certain threshold, its incremental contribution to self-efficacy may diminish [27]. Thus, higher levels of knowledge do not necessarily translate into greater confidence in managing health behaviors. In addition, the relatively low internal consistency of the Hypertension Knowledge Scale (KR-20=.51) may have attenuated associations involving hypertension-related knowledge. This low reliability may be attributable to the multidimensional structure of the instrument, which could have led to inconsistent responses [45]. Therefore, associations involving hypertension-related knowledge should be interpreted with caution because they may underestimate the true relationship between knowledge and self-efficacy.

Second, eHealth literacy was not directly associated with health-promoting behaviors, and its total effect was not statistically significant. However, eHealth literacy had a significant indirect effect through self-efficacy in chronic disease management. This finding partially differs from previous studies reporting direct associations between eHealth literacy and health-promoting behaviors [22,34], whereas the indirect association through self-efficacy is consistent with previous findings [18]. These results may be explained by contextual and sample-specific factors. Social support showed strong associations with both health-promoting behaviors and self-efficacy, which may have attenuated the relative contribution of eHealth literacy in the model. In addition, online data collection may have led to overrepresentation of individuals familiar with digital environments, resulting in relatively high eHealth literacy and restricted variability. This restriction of range may have attenuated the association between eHealth literacy and health-promoting behaviors.

Further examination of the eHealth literacy subdomains supports this interpretation. Functional eHealth literacy, which reflects basic reading and writing skills for accessing online health information, was the highest subdomain score, whereas critical and communicative eHealth literacy scores were lower [34]. Although participants appeared able to access information, their ability to critically interpret information and apply it to health-related action may have been more limited. Accessing and repeatedly searching for information may be insufficient to facilitate behavioral change because information overload and variable information quality can hinder integration of knowledge into decision-making and practice [48]. In this context, eHealth literacy may function as a supportive resource through indirect pathways rather than as a primary determinant of behavior. However, given the cross-sectional design, these interpretations should be considered associative rather than causal. Future studies using larger and more diverse samples are warranted to replicate these findings and further examine the differential roles of eHealth literacy subdomains in health-promoting behaviors.

Third, health attitudes were not significantly associated with health-promoting behaviors among middle-aged women with hypertension. This result differs from previous studies reporting a direct association between health attitudes and health-promoting behaviors in middle-aged women [15] and individuals with mental illness [40]. However, the present result is consistent with studies indicating that health attitudes were not directly associated with lifestyle modification [1] or self-care behaviors [9] in patients with hypertension.

This finding may be interpreted in light of the sociocultural context of middle-aged Korean women, who have traditionally assumed primary responsibility for children’s education, family health management, and extended family relationships. These responsibilities may lead women to prioritize family members’ needs over their own and to adopt a family-centered orientation in daily life [8]. Another possible explanation is that middle-aged women may experience negative psychosocial factors, such as perceived stress or body image dissatisfaction, and these factors may be associated with lower engagement in health-promoting behaviors [7,28]. Although the present study did not directly measure these variables, such psychosocial factors may have influenced the relationship between health attitudes and health-promoting behaviors. Future studies should examine this relationship while including psychosocial variables such as perceived stress and negative emotional states.

Health attitudes were indirectly associated with health-promoting behaviors through self-efficacy in chronic disease management. Individuals with positive health attitudes may have difficulty translating these attitudes into action when self-efficacy is low, whereas higher self-efficacy may be associated with greater engagement in health-promoting behaviors regardless of attitude level [26]. These findings indicate that self-efficacy may be an important pathway for understanding health behavior among middle-aged women with hypertension.

Fourth, social support was significantly associated with health-promoting behavior among middle-aged women with hypertension. This finding is consistent with previous studies showing that greater social support is associated with higher levels of health-promoting behaviors in patients with hypertension [23]. Women with hypertension who receive support from family members and friends tend to engage more frequently in physical activity [9] and maintain self-management behaviors more effectively when supported by family- and community-based interventions [49]. Accordingly, approaches that incorporate relational and community-based support structures may warrant further investigation. Future research is needed to examine whether such support is associated with sustained engagement in health-promoting behaviors in this population.

Self-efficacy in chronic disease management partially mediated the relationship between social support and health-promoting behaviors, suggesting that social support may be linked to behavioral engagement both directly and indirectly through self-efficacy. This result is consistent with previous research showing direct and indirect associations between social support and health-promoting behaviors through self-efficacy [26]. These findings suggest that middle-aged women with hypertension who have limited social support, including those living alone, may be particularly vulnerable to lower engagement in health-promoting behaviors. Therefore, future studies should explore strategies to support self-efficacy in chronic disease management, particularly for individuals with limited relational resources. For example, health education and individualized lifestyle counseling in community healthcare settings, combined with nurse-led follow-up support in outpatient settings, may help promote sustained engagement in health-promoting behaviors [1]. In addition, ongoing communication with nurses, including encouragement and support, as well as achievable incremental goals and reinforcement through repeated success experiences, may help enhance self-efficacy [28] and facilitate engagement in health-promoting behaviors in this population [6].

Finally, self-efficacy in chronic disease management was significantly associated with health-promoting behaviors among middle-aged women with hypertension. This result is consistent with previous studies showing that self-efficacy is associated with health-promoting behaviors in patients with hypertension [24] and middle-aged women with obesity [28]. Patients may strengthen their sense of efficacy by sharing successful experiences of blood pressure control through adherence to health behaviors, setting short-term behavioral change goals, and monitoring and recording their progress in collaboration with nurses [6,23]. In this context, self-monitoring and feedback from healthcare providers may be useful strategies for supporting self-efficacy in chronic disease management.

This study has several limitations. First, a cross-sectional design with convenience sampling through an online survey was used, and selection bias related to online recruitment cannot be ruled out. Participants may have been more familiar with digital environments, and individuals without adequate internet access, particularly those from socioeconomically disadvantaged backgrounds, may have been underrepresented. Accordingly, the findings should be interpreted with caution and may not be generalizable to all individuals with hypertension. Second, the relatively low internal consistency of the Hypertension Knowledge Scale (KR-20=.51) is a methodological limitation. This low reliability may have attenuated associations involving hypertension-related knowledge and led to underestimation of its relationships with other variables in the model. Future studies using larger and more diverse samples, along with additional validation approaches, are needed to strengthen the reliability and validity of this instrument. Third, although the overall fit indices met commonly accepted criteria, the RMSEA (.08) and AGFI (.82) values suggest that model fit was marginal rather than optimal, indicating that the proposed model did not fully correspond to the observed data. However, because excessive model modification to improve fit may compromise theoretical validity and meaningful interpretation, the hypothesized model was retained without additional modifications. Therefore, the model may not provide a comprehensive representation of health-promoting behaviors in this population, and the structural associations should be interpreted with caution.

Despite these limitations, this study contributes to understanding health-promoting behaviors among middle-aged women with hypertension by applying the IMB framework within a socio-relational context. The findings suggest that strengthening social support and self-efficacy may be a useful direction for future intervention research aimed at promoting health behavior change in this population. Further longitudinal and experimental studies are needed to clarify these relationships and identify effective strategies for supporting sustained health-promoting behaviors among middle-aged women with hypertension.

## Figures and Tables

**Table 1.** General characteristics of participants (N=331)

Characteristics	Categories	n (%)	Mean±SD
Age (year)	40 to <50	97 (29.4)	53.34±6.29
	50 to <60	165 (49.8)	
	60 to <65	69 (20.8)	
Cohabitation status	Living alone	26 (7.9)	
	Living with family	305 (92.1)	
Educational level	≤Middle school	4 (1.2)	
	High school	109 (32.9)	
	≥College	218 (65.9)	
Employment status	Employed	242 (73.1)	
	Not employed	89 (26.9)	
Monthly household income (×10,000 KRW)	<200	25 (7.5)	
	200 to <300	36 (10.9)	
	300 to <400	53 (16.1)	
	400 to <500	68 (20.5)	
	≥500	149 (45.0)	
Duration of hypertension (year)	<6	165 (49.8)	7.70±6.63
	6 to <11	82 (24.8)	
	11 to <16	48 (14.5)	
	16 to <21	15 (4.5)	
	≥21	21 (6.4)	
Taking antihypertensive medication	Yes	304 (91.8)	
	No	27 (8.2)	
Comorbidities (duplicate responses possible)	None	91 (27.5)	
	Diabetes mellitus	47 (14.2)	
	Hyperlipidemia	158 (47.7)	
	Cancer	11 (3.3)	
	Kidney disease	7 (2.1)	
	Cardiovascular disease	8 (2.4)	
	Musculoskeletal disease	62 (18.8)	
	Digestive disease	39 (11.8)	
	Others^†^	9 (2.7)	
Body mass index (kg/m^2^)	Underweight (<18.5)	4 (1.2)	24.69±4.19
	Normal weight (18.5 to <23)	130 (39.3)	
	Pre-obese (23 to <25)	61 (18.4)	
	Obese (class I) (25 to <30)	97 (29.3)	
	Obese (class Ⅱ) (30 to <35)	35 (10.6)	
	Obese (class Ⅲ) (≥35)	4 (1.2)	
Subjective health status	Unhealthy	123 (37.1)	
	Moderate	145 (43.8)	
	Healthy	63 (19.1)	
Menstruation	Regular	71 (21.5)	
	Irregular	53 (16.0)	
	Menopause	207 (52.5)	51.42±4.38
	Surgical menopause	33 (10.0)	
	Natural menopause	174 (52.6)	
Drinking (time)	≤1/month	242 (73.1)	
	3 to 4/month	53 (16.0)	
	1 to 2/week	24 (7.3)	
	≥3/week	12 (3.6)	
Smoking	Yes	19 (5.7)	
	No	312 (94.3)	

KRW: Korean won (one million KRW is roughly 700 US dollars).

† Respiratory and thyroid disease.

**Table 2.** Descriptive statistics and factor loading of confirmatory factor analysis (N=331)

Latent variables	Measurement variables	Mean±SD (score^†^)	Cronbach’s α	Range	Skewness	Kurtosis	β	SE	C.R. (*p*)	CR	AVE
Hypertension-related knowledge		17.86±1.60	.51^‡^	11.0–20.0	–0.81	0.60	.90				
eHealth literacy	Functional	4.03±0.53	.93	2.38–5.00	–0.11	0.24	.77	.08	12.14 (<.001)	.99	.97
	Communicative	3.46±0.76	.94	1.27–5.00	–0.30	–0.04	.73	.11	11.85 (<.001)		
	Critical	3.51±0.57	.91	1.83–5.00	–0.30	0.47	.78				
Health attitudes	Feeling	3.86±0.52	.64	2.60–5.00	–0.18	–0.41	.61	.07	9.67 (<.001)	.99	.97
	Belief	3.78±0.56	.64	1.00–5.00	–0.88	2.56	.75	.08	11.16 (<.001)		
	Intention to act	3.79±0.61	.86	1.00–5.00	–0.43	1.17	.76				
Social support	Healthcare provider	2.69±0.91	.94	1.00–5.00	0.11	–0.34	.53	.10	8.33 (<.001)	.97	.92
	Family	3.90±0.66	.90	1.00–5.00	–0.54	0.80	.61	.07	9.45 (<.001)		
	Friends	3.52±0.73	.88	1.00–5.00	–0.24	–0.13	.80				
Self-efficacy in chronic disease management	Symptom management	6.58±1.64	.97	1.50–10.00	–0.53	0.09	.87	.06	16.72 (<.001)	.94	.89
	Health behavior	7.06±1.54	.91	2.00–10.00	–0.53	0.03	.92				
Health-promoting behavior	Health responsibility	2.41±0.63	.85	1.00–4.00	0.09	–0.21	.73	.07	15.29 (<.001)	.99	.98
	Physical activity	2.46±0.78	.91	1.00–4.00	–0.04	–0.36	.61	.09	12.17 (<.001)		
	Nutrition	2.63±0.63	.77	1.00–4.00	–0.02	–0.23	.64	.08	12.91 (<.001)		
	Spiritual growth	2.66±0.56	.89	1.22–4.00	–0.08	–0.29	.89	.06	21.09 (<.001)		
	Interpersonal relations	2.74±0.51	.87	1.25–4.00	–0.05	–0.15	.87	.05	20.26 (<.001)		
	Stress management	2.52±0.49	.79	1.38–3.88	0.12	–0.32	.85				

AVE: Average variance extracted; β: standardized estimate; C.R.: critical ratio; CR: construct reliability.

† Hypertension-related knowledge was analyzed using total scores, whereas all other variables were converted to mean scores. Possible score ranges: hypertension-related knowledge (0–20), eHealth literacy (1–5), health attitudes (1–5), social support (1–5), self-efficacy in chronic disease management (1–10), and health-promoting behaviors (1–4);

‡ Kuder–Richardson 20.

**Table 3.** Parameter estimation results of the structural model (N=331)

Endogenous variables	Exogenous variables	SMC	Standardized direct effect (*p*)	Standardized indirect effect (*p*)	Standardized total effect (*p*)
Self-efficacy in chronic disease management	Hypertension-related knowledge	42.6	–.01 (.806)	-	–.01 (.806)
	eHealth literacy		.23 (.012)		.23 (.012)
	Health attitudes		.32 (.017)		.32 (.017)
	Social support		.26 (.006)		.26 (.006)
Health-promoting behavior	Hypertension-related knowledge	60.4	.11 (.024)	.00 (.802)	.11 (.024)
	eHealth literacy		.07 (.418)	.06 (.018)	.12 (.156)
	Health attitudes		.17 (.125)	.08 (.023)	.24 (.048)
	Social support		.43 (.001)	.07 (.012)	.49 (.001)
	Self-efficacy for managing chronic disease		.25 (.007)	-	.25 (.007)
Fit indices for hypothetical model	*χ*²=400.42, *χ*²/df=3.31, AGFI=.82, CFI=.90, IFI=.91, RMR=.03, SRMR=.05, RMSEA=.08				

AGFI: Adjusted goodness-of-fit index; CFI: comparative fit index; IFI: incremental fit index; RMR: root mean square residual; RMSEA: root mean square error of approximation; SMC: squared multiple correlation; SRMR: standardized root mean square residual.
